# Perceptions of and Experiences with Consumer Sleep Technologies That Use Artificial Intelligence

**DOI:** 10.3390/s22103621

**Published:** 2022-05-10

**Authors:** Esther Oh, William Kearns, Megan Laine, George Demiris, Hilaire J. Thompson

**Affiliations:** 1 Biobehavioral Nursing and Health Informatics, School of Nursing, University of Washington, Seattle, WA 98195-7266, USA; oh.esther.s@gmail.com (E.O.); kearnsw@uw.edu (W.K.); mlaine@uw.edu (M.L.); 2 Schools of Nursing and Medicine, University of Pennsylvania, Philadelphia, PA 19104, USA; gdemiris@upenn.edu

**Keywords:** adults, sleep, artificial intelligence, user-centered design

## Abstract

This study aims to assess the perspectives and usability of different consumer sleep technologies (CSTs) that leverage artificial intelligence (AI). We answer the following research questions: (1) what are user perceptions and ideations of CSTs (phase 1), (2) what are the users’ actual experiences with CSTs (phase 2), (3) and what are the design recommendations from participants (phases 1 and 2)? In this two-phase qualitative study, we conducted focus groups and usability testing to describe user ideations of desires and experiences with different AI sleep technologies and identify ways to improve the technologies. Results showed that focus group participants prioritized comfort, actionable feedback, and ease of use. Participants desired customized suggestions about their habitual sleeping environments and were interested in CSTs+AI that could integrate with tools and CSTs they already use. Usability study participants felt CSTs+AI provided an accurate picture of the quantity and quality of sleep. Participants identified room for improvement in usability, accuracy, and design of the technologies. We conclude that CSTs can be a valuable, affordable, and convenient tool for people who have issues or concerns with sleep and want more information. They provide objective data that can be discussed with clinicians.

## 1. Introduction

An estimated 50–70 million adults in the United States have a sleep disorder [1]. Such disorders include sleep deprivation, snoring, insomnia, obstructive sleep apnea, and narcolepsy. Lack of a healthy amount of quality sleep and various sleep disorders have been associated with an increased risk of hypertension, obesity, diabetes, and cardiac disease1 as well as decreased cognitive performance, decreased quality of life, increased risk of injury, and a higher risk for mortality [2]. Americans spend billions of dollars annually on sleep-related clinic visits, health care services, and medications.1 The United States loses an estimated 1.23 million working days a year due to the lack of sleep, which is an additional economic loss of $280 to $411 billion a year [3].

The emergence of new technologies has introduced ways to measure aspects of sleep outside of clinical or laboratory settings. Consumer Sleep Technologies (CSTs) are applications, wearables, tracking pads, and other devices used to measure and improve sleep outside of hospital settings [4,5]. All CSTs referred to in this study use artificial intelligence (AI) which involves the use of computer systems to simulate human intelligence, such as pattern detection, learning and/or actions to assist with human health. These systems will be further referred to as “CSTs+AI”. Such CSTs have the goal to detect and stop snoring, track sleep duration, assess sleep quality, monitor vital signs, and/or detect teeth grinding, providing the first step toward automated expert-level analysis of sleep patterns from home. In contrast, CSTs that do not use AI are mostly focusing on logging sleep diary data, visualizing self-reported ratings of sleep, or providing educational tips for improved sleep hygiene. “Usability” for this study is defined as the quality of the user experience and degree of effectiveness and efficiency in the use of a tool. By assessing user perceptions of CSTs+AI, evaluating usability, and identifying ways to improve such tools, CSTs+AI can be enhanced, created, and utilized to promote better sleep health and improve sleep disorders.

### 1.1. Aims

The purpose of this project is to understand user perspectives of CSTs+AI. Data collection for this study consisted of two phases. The first phase included focus groups demonstrating different CSTs to understand users’ thoughts about a suite of CSTs that use AI and identify ideations on desires and hopes for CST designs. The second phase consisted of at-home usability testing of three CSTs: a headband, a sleeping mat, and a mobile application. After using these technologies for five nights, they were interviewed about their experiences with them. The aims of this study are to answer the research questions: (1) what are user perceptions and ideations of CST+AIs (Phase 1), (2) what are user experiences with CSTs+AI (Phase 2), (3) and what are design recommendations from users (Phases 1 and 2)?

### 1.2. Significance

Mobile applications and tools can be conveniently used at home and may facilitate more cost-effective assessment than that in sleep studies. Findings from this study can be applied to at-home tools. As CSTs continue to evolve, information from user ideation and experience research can bring beneficial insight to improve these tools. Improved tools and technologies can promote sleep quantity and quality for those living with sleep disorders. We must understand how to help consumers and their needs and preferences in order to reduce sleep deprivation and sleep disorders and promote sleep health.

### 1.3. Current Use of Consumer Sleep Technology

A survey of 1029 adults in the U.S. found that 22% of consumers had owned or used some sleep technology [6]. Men were more likely to use CSTs and 71% of CST users were under 45 years old. Less than 10% of survey respondents reported that they regularly use sleep technology. Those who used sleep technology were more likely to have healthier habits and be active participants in their health. Among the participants who used CSTs, 59% were satisfied with current sleep technology. Reported barriers to using CSTs were cost and anticipated discomfort with wearable CSTs. Among the participants who did not use sleep technology and slept less than six hours a night, 32% thought that CSTs would help them be healthier. Twenty-nine percent of respondents said that they were not aware of sleep technology [6].

### 1.4. Accuracy of Consumer Sleep Technology

Despite the increasing recognition of the potential benefits of CSTs, there is a lack of information about their validity, accuracy, and reliability. The Food and Drug Administration does not regulate CSTs, making it more difficult to reach a consensus on standardization. Additionally, the lack of information about validity and accuracy in CSTs makes it difficult for providers to trust and use the data and information recorded and produced [6]. Recent studies have shown that newer commercial devices are performing better than earlier models [7,8].

Polysomnography (PSG) is the gold standard for evaluating sleep and is used in clinical sleep studies. [9] While earlier studies comparing CSTs to PSG showed poor correlation challenging the utility of CSTs as clinical tools [9], more recent work demonstrated that CSTs may have the potential for detecting outcomes beyond binary sleep-wake although inconsistencies in findings indicate that further validation work against PSG is needed [10,11]. Additionally, most studies test CSTs in controlled environments. A recent study, however, tested the performance of CSTs under naturalistic unrestricted home sleep conditions and compared them to PSG; findings indicated that CSTs were best utilized for tracking sleep-wake outcomes and not sleep stages in real-world conditions [12].

Overall, there remain challenges for the scientific community to validate CSTs given the time-consuming nature of conducting these studies and the quick pace of change in CSTs [8]. Although CSTs are not currently approved for clinical purposes or diagnosis of any conditions, they may complement traditional PSG data. CSTs can provide a longer-term assessment with subjective information [13].

### 1.5. Known Advantages and Disadvantages of Consumer Sleep Technology

CSTs include mobile applications, wearables, embedded CSTs, desktop or website CSTs, and accessory appliance platforms. Advantages to mobile applications are convenience, ease, capability, and accessibility. Disadvantages include reduced processing power, light, and noise, which can interfere with sleep. Wearable CSTs use attachments or sensors on the body or clothing and track movements and biometric information. Sensors may strengthen accuracy due to physical contact but may introduce challenges, such as discomfort, short battery life, the potential for sensor damage, and dislodgement during sleep. Finally, embedded CSTs, such as a tracking pad or smart mattress, are unobtrusively concealed in the sleep environment but can require more space and cost more.

Mobile applications are the most popular type of CST and are used for sleep tracking, alarms, education, recording sounds, and dream logging [5,14]. However, one survey showed that half of the respondents who downloaded mobile health applications discontinued using the applications due to poor user experience, lack of engagement, and hidden costs [15]. Respondents who did not use mHealth applications at all were concerned about data collection and cost. Some features that respondents wanted were alarms to help them wake up and stay awake, audio features, encouragement, recommendations, social features, sleep tracking, behavior tracking, and data visualization. Usability, intuitiveness, and bug-free designs were common themes for improvements with sleep applications.

## 2. Materials and Methods

### 2.1. Study Design

This study consisted of two phases: Phase 1—focus groups to understand the needs and desires of individuals with sleep disturbance related to CST, and Phase 2—a usability study of CSTs with persons with sleep disturbance. The COVID-19 pandemic necessitated changes to the study design to move focus groups to an online format and conduct the usability study remotely.

#### 2.1.1. Consumer Sleep Technologies That Use Artificial Intelligence

The CSTs included in this study were selected through an exhaustive search of AngelList, Google, and Amazon for companies and products that referenced the terms “artificial intelligence” and “sleep” at the end of 2019. We chose these websites for the search to capture CST innovations across a spectrum from idea to startup/incubation to licensed products. This search returned the following device types: head-mounted devices, tracking pads, standalone mobile applications, and responsive devices (Table 1). We excluded mattresses from the sleep tracking pad category for our study due to practical concerns over storage and shipping to participants.

##### Head-Mounted Devices

Head-mounted devices (HMDs) use straps or adhesive pads to place one or more sensors in contact with an individual’s forehead. All devices in this category include sensors for accelerometry, pulse oximetry, and measuring respiration. The more expensive HMDs also capture electroencephalographic (EEG) signals for sleep stage prediction. The following devices were included in our study in this category: Beddr (Hancock Medical, Inc.), Dreem Headband (Dreem), and Muse S (InteraXon Inc.).

##### Tracking Pads

This category includes stand-alone tracking pads and smart mattresses with integrated sensors to measure body position, heart rate, and detect breathing disturbances including snoring. The focus groups and usability study included one sleep tracking pad, the Sleep Analyzer (Withings), which is placed under a mattress and syncs with a mobile application that provides feedback and coaching. The tracking pad in this study was unique in that it only required a single time setup.

##### Standalone Mobile Applications

Standalone mobile applications use machine learning and sensors commonly found on smartphones to track and assess sleep, detect snoring, predict sleep cycles from body position, wake users with an alarm, and/or provide audio to enhance the sleep environment. The following devices were included in this category: Do I Snore or Grind (SleepScore Labs), Sleep Cycle (Sleep Cycle AB), Sleeprate (Sleeprate), and Sleep Score (SleepScore Labs). Sleep Cycle support states that the app can differentiate sounds between bed partners if both bed partners are using the app and connected to the same WiFi [16].

##### Responsive Devices

While the other devices on this list use their sensors for passive tracking, others intervene when detecting sleeping disturbances. These devices detect disturbances and actively adjust the user’s body position to address the disturbance. One device in this category is the Smart Nora (Smart Nora, Inc.), which detects snoring and changes the head-position of the user to open their airway and stop snoring.

#### 2.1.2. Screening Criteria

For both phases, participants who responded to an advertisement on a social media network were screened through a REDCap (Vanderbilt University) survey approved by the University of Washington Institutional Review Board (STUDY00009791). Participants were required to be at least 18 years old, sleep at night, have English proficiency, physical and cognitive ability to use the devices, and a Pittsburgh Sleep Quality Index (PSQI) score of more than five indicating poor sleep quality [17]. All participants provided informed consent to participate including the digital recording of focus groups or interviews.

In phase 2, participants in the usability study were also required to own an iPhone 6 (Apple Inc.) with iOS 11.3 or newer due to the compatibility with the HMD.

#### 2.1.3. Phase 1-Focus Groups

In the first phase, the study team conducted four online focus groups (Appendix A). A study team member played short video demonstrations for each of the proceeding technologies to guide discussion for each device category: Beddr (Hancock Medical, Inc.), Dreem Headband (Dreem), and Muse S (InteraXon Inc.), Sleep Analyzer (Withings), Smart Nora (Smart Nora), Do I Snore or Grind (SleepScore Labs), Sleep Cycle (Sleep Cycle AB), Sleeprate (Sleeprate), and Sleep Score (SleepScore Labs).

**Figure 1:**
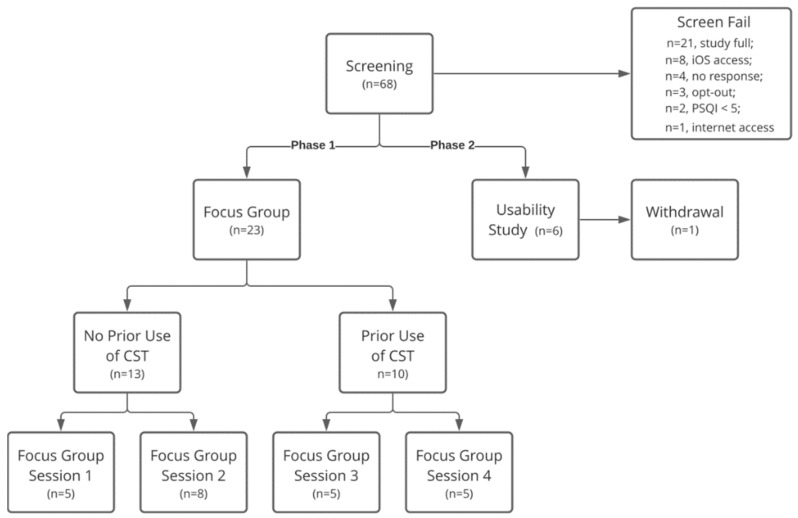
Study flow diagram of participants in focus groups.

To encourage open discussion, participants in the focus group study were split into two cohorts based on whether they had prior use of CSTs or had no prior experience with CSTs. In total, four one-hour focus group sessions were held over Zoom video conferencing software (Zoom Video Communications, Inc.). For each session, a research team member provided a multi-media demonstration of each device and another research team member moderated a structured discussion about the following device groups: head-mounted devices, tracking pads, responsive devices, and standalone mobile applications. The flow diagram of criteria for focus groups and usability study is shown in Figure 1.

#### 2.1.4. Phase 2-Usability Testing

In the second phase, a group of six participants was invited to try a subset of these devices in their homes before taking part in a scripted interview. The subset included: Beddr (Hancock Medical, Inc.), Sleep Cycle (Sleep Cycle AB), and Sleep Analyzer (Withings).

The HMD evaluated in the usability study consisted of an optical sensor and accelerometer adhered to the forehead and was cleared by the FDA to measure sleep duration, oxygen levels, heart rate, sleep positions, and stopped breathing events. The total weight of the device is approximately six grams and is held in place by hypoallergenic medical-grade, single-use adhesives. The HMD connects to a mobile application that shows trends and sleep quality scores.

The mobile application evaluated in the usability study monitored sleeping patterns, used sound analysis to track sleep states, monitored movements, woke users during the lightest sleep phase, and evaluated sleep quality. The user’s phone is placed either next to the bed or on the mattress and the application uses a built-in microphone or accelerometer to analyze movements. The application also detects snoring, sleep talking, and other sounds. It is recommended that the phone is plugged into a charger during use because of battery usage.

Usability testing and semi-structured interviews were used to assess different AI sleep technologies and identify how these technologies could be improved. The snoring device was not evaluated in the usability testing because the inclusion criteria did not include snoring. Once participants were selected, the study team participants were consented and provided with instructions on how to use the CSTs. Participants used the Beddr, Sleep Cycle App, and Withings devices at home for five nights, two of which were weekend nights. After the use of all CSTs, the participants met with the study team for a semi-structured interview. The interview guide includes questions focused on what participants would use CSTs for, beneficial features of the tested CSTs, how the tested CSTs could be improved, and what participants want from future CSTs (Appendix A). Participants were compensated in the form of a $50 gift card upon interview completion and return of all devices.

### 2.2. Qualitative Content Analysis

The study team manually edited Zoom-generated transcripts and applied thematic analysis to the focus group and interview recordings. The codebook (Appendix B) was developed through an iterative process of consensus building between three study team members (EO, WK, ML). For the focus groups, two annotators coded each participant’s utterance separately with disagreement arbitrated by a third annotator. For the usability testing interviews, disagreements were resolved through consensus as only two annotators reviewed the transcripts.

## 3. Results

### 3.1. Phase 1-Focus Groups

#### 3.1.1. Sample

PSQI scores ranged from 5 to 18. Participant ages ranged from 25 to 68 years old, with an average age of 41.

#### 3.1.2. General Themes

Three major themes from the analysis include: (1) participants prioritize comfort, actionable feedback, and ease of use, (2) participants are enthusiastic that sleep self-management tools may provide more customized advice and utility than traditional PSG, and (3) participants are most interested in adopting or trying new tools that integrate with the existing products and strategies they use to improve sleep. Exemplar quotes of these themes from focus groups participants are referenced by their group and participant number (FG#, P#).

Participants envisioned how AI would work in the CSTs: “*I think it will be able to kind of like diagnose like your sleeping patterns and maybe just recommend things that you could do to kind of better it overall*” (FG2, P1). Another imagined a diagnostic tool but expressed concerns about accuracy: “*you essentially like plug in a bunch of symptoms and it shoots out like a really high-tech version of Google medical whatever it’s called. Um, but I’m not entirely certain how effective it is*” (FG2, P5).

#### 3.1.3. Consumer Priorities

Among the demonstrated CSTs+AI, participants valued comfort. “*It’s really hard to discern what’s different about any of these things except for the execution. Do I want to sleep on a pad? Do I want a thing on my head? Do I want a thing on my body? I’m going to go with the less the least intrusive device*” (FG1, P2). Another participant described: “*I like the one that is not invasive goes under the mattress…I definitely don’t like the one with the adhesive. There’s no way I can take it on and off*” (FG4, P3). The few participants who prioritized accuracy from CSTs+AI were willing to sacrifice personal comfort. Some participants also desired actionable feedback: “*We don’t really need a score to tell us if we slept well. We already know if we slept well. So unless there’s something unusual that it can say hey look, you’re not getting enough REM sleep, go look at that with your doctor. I don’t know that it will be all that helpful in actually changing anything for you*” (FG3, P2).

#### 3.1.4. Desires and Suggestions

Participants were excited about the possibility of receiving customized feedback relevant to their habitual sleeping environment. For example, they envisioned that CST+AI might provide information about reasons for poor sleep: “*Oh, you were, you know, moving around a lot at 2am and but it was also noisy outside and then I would have insight as to why*” (FG1, P2). Another participant envisioned how a CST+AI could “*look at the space in the room where you’re sleeping and determine like how good these settings in this room are for your sleep like it could look at the temperature, it could look at the light*” (FG4, P1).

#### 3.1.5. Comparisons with Habitual Sleep Strategies and Technologies

Participants typically compared the demonstrated CSTs+AI with their existing sleep self-management strategies and products. One participant liked that a demonstrated app was similar to their smart watch: “*I could … look on to the computer or my phone or whatever and [it can] tell me if you know how I slept the day before… So I just think it’s a lot easier and more convenient to me to have something like this*” (FG2, P4). Another participant described: “*I would like to have an app that I could use that I could schedule, you know, to have the white noise in the background… [it] would not be sending me notifications and needing me to update it constantly and… [it] wouldn’t run down my battery like crazy either*” (FG3, P5). Further comparisons were made with virtual assistant AI technology: “*You can set a skill on your Alexa to do the same thing every night … it kind of helps me calm down and wind down my day and the same thing, the routine of the same thing every night when I can get in bed on time*” (FG4, P5).

### 3.2. Phase 2-Interviews

#### 3.2.1. Sample

For the usability test sample, PSQI scores ranged from 10 to 16. Participant ages ranged from 24 to 71 years old, with an average age of 42.

All usability study participants completed the interview and are referenced as Study Participant 1–6 (SP 1–6). Of the six participants, five participants tried all technologies. The sixth participant withdrew from the study after trialing the mobile app and reported that the awareness of data collection aggravated their anxiety around sleep.

#### 3.2.2. General Themes

All six participants had prior experience with at least one CST. Reasons participants gave for discontinuing use prior to the study included cost, the aggressiveness of goals, and limited device storage. SP 2 stated that they “*always end up deleting [sleep mobile applications] off [their] phone because they just take up room and [they] don’t use them for a while*”. When asked about their expectations of the three technologies, some participants expected the CSTs to track sleep duration and cycles and to be able to sleep better. SP 2 said they would use sleep technologies “*to get a better understanding of [their] sleep habits and to try to stabilize [their] sleep and bedtime routine*”. Users wanted a better understanding of their sleep patterns and how long was spent in different sleep cycles.

Participants stated that CSTs+AI help provide an accurate picture of the quantity and quality of sleep. With a better understanding of their sleep, users can change sleep habits and behaviors to improve sleep. SP 4 stated that “*once you have the information from the first few nights, you know a few nights of it, you then have to take the next steps into something that’s going to change your sleep behavior*”. The top feature participants wanted in a CST+AI is information about sleep cycles, (i.e., a *sleep score)*. Other features that the vast majority of participants would like are graphs/visuals, interaction from apps, ease of use, aids to help with falling asleep, and alerts of any detected abnormalities.

SP 4 compared her experiences with the CSTs+AI with their previously completed sleep studies: they had “*essentially the same information, [and] it was a lot easier*”. SP 4 stated that the CSTs+AI were useful if you did not have any information about your sleep prior to use. Other participants deferred clinical sleep studies in the past due to insurance or cost issues.

SP 1 stated that they would continue using the sleep tracking pad and felt it was the most accurate of the three trialed technologies. SP 2, SP 3, and SP 4 would continue using the Sleep Cycle app. SP 2 would continue using the app because “*it did give … so many options of how to make it more your own*”. SP 4 stated that they would continue using the app because “*it reminded you of your bedtime*”. SP 5 would continue using both the sleep tracking pad and mobile application.

### 3.3. Head-Mounted Devices

Four of five usability study participants expressed that the adhesives were not strong enough to keep the device secured on the forehead. However, none found the device to be uncomfortable. SP 5 said they used different skincare products, but “*would just wake up and it’s like way across the room*”. SP 4 said that even though the device did not adhere well, they felt the graphs and recordings were accurate. Two users stated that they found putting a new adhesive on every night to be tedious. Users wanted a stronger adhesive that would stay on overnight and an adhesive that would be easier to apply.

### 3.4. Standalone Mobile Applications

The mobile application evaluated in the usability study was noted to be the “*favorite*” amongst participants due to its claim to wake the sleeper during their lightest sleep state within a personalized wake-up window. In the interviews of usability study participants, three participants mentioned that they enjoyed the ability to personalize the alarm and music on the application. SP 2 liked that they “*could set [the] alarm and then it would find the best time to wake [them] up within [their] sleep cycle within a parameter of time*”. Four users also enjoyed that the application recorded noises, such as snoring, coughing, and talking. However, there was difficulty in differentiating noises among users, bed partners, pets, and others. SP 2 said that “*it picked up [their] cat purring like it said [they were] snoring*”. 

### 3.5. Tracking Pads

During the usability study, three participants stated that the sleep tracking pad was easy to use because they did not have to think about it once it was set up. However, two participants expressed the desire to have a way to turn on the pad or know when the pad was recording. SP 1 said that “*it’s nice not having to start it, but since it was kind of tracking before [they] went to sleep, it might be nice to get it to start tracking when [they] wanted it to*”. Sometimes participants would watch television or read in their beds and the pad would record data. Placing the pad under the mattress and rearranging it to accommodate an outlet for the mat was difficult, but once the pad was in place, participants noted the ease of use. SP 5 said that they expected the pad to be more expensive than the stated purchase cost and felt the data from the technologies could be useful to clinicians.

## 4. Discussion

In phase I, there were no significant differences in themes that arose throughout the two types of focus groups based on previous use of CSTs, and participants primarily focused on positive examples of AI. Participants stated that they could discuss the information provided by CSTs+AI with their health care providers to learn about and diagnose sleep disorders. The cost and inconvenience of PSGs increase the interest in and use of CSTs+AI [13]. CSTs+AI could overcome some limitations of PSGs, such as a foreign sleep environment and data captured only from one night of sleep. Having only one night of information can limit the translatability of PSG studies to real life [18]. Sleep changes from night to night and longitudinal data can provide valuable information on trends. Although CSTs cannot provide medical advice and need more testing, they can provide some objective data for people with sleep disorders or suspected sleep disorders [19]. Those with sleep disorders or difficulties with sleep may have subjective information, but objective data can have more utility. This information from the technologies can be discussed with clinicians to help make more informed decisions about care and if additional tests or studies are warranted. Focus group participants expressed interest in sleep self-management tools that could provide more personalized feedback and utility than PSGs. This sentiment was echoed in the usability testing as SP 4 stated that the CSTs+AI in the study provided information from PSGs in a more accessible way. There is a need to integrate CSTs with evidence-based sleep self-management tools to address this concern. Commercially available tools generally provide general feedback that is not tailored to the individual. Leveraging AI in a more robust manner could address this need given personal differences in what constitutes optimal sleep (e.g., amount, duration, timing, location).

When patients present CST data to clinicians, there may be disagreements on how patient-generated health data (PGHD) should be used [20,21]. One systematic review found that PGHD improved clinicians’ understanding of patients’ health, but clinicians had a varied interest in PGHD and encountered barriers to its use in clinical care, such as time constraints, lack of workflow integration, information overload, and lack of reimbursement [20]. PGHD has the potential to improve patient-clinician collaboration, patient care, and outcomes; however, clinicians may not be familiar with all the different technologies and how to utilize the information. Patients may have expectations of how clinicians utilize PGHD, which may not be met and could cause strain on the patient-clinician relationship. It is important that the technologies support clinical workflow and integration and present clinicians with information in a clear manner that is easy to understand. Some clinicians are concerned with the validity and reliability of the data of PGHD [14,21]. PGHD needs to be reliable to help clinicians make decisions. CST companies should consider how clinicians will use the data. Further studies that incorporate clinicians as the end users of the data are needed to see if and how this can be integrated into usual care. Given the geographic dispersion and overall lack of available sleep specialists, it would also be important to identify if primary care providers are able to use this data to improve population health. This may enable such technologies to address health disparities related to sleep.

It is important for CSTs+AI to perform as they are intended and marketed. Four of five usability study participants stated that the HMDs did not stay adhered throughout the night, which presented issues with use, data collection, and accuracy. In the focus groups, some participants expressed concerns regarding accuracy in differentiating between the user and other bed partners and pets. This was confirmed in the usability testing. Future iterations of snoring detecting features could consider how to improve accuracy with multiple noise sources. The design of the sleep tracking pad could be improved as some participants had issues with having the pad constantly plugged into the charger and having to rearrange bedroom furniture to accommodate the pad.

Although usability participants had differing opinions on each trialed device, five of the six participants agreed that CSTs had utility and that they would continue using at least one. Participants from both the focus groups and usability study were interested in continuing the use of CSTs+AI introduced in the study. It is important for CST companies to be aware of users’ experiences and recommendations so that CSTs can be enhanced to improve the quantity and quality of sleep for those living with sleep disorders. One participant withdrew from the study after using a single technology, because thinking about sleep while using the technology increased anxiety around sleep. This calls for a consideration of how altering sleep environments can affect sleep.

### 4.1. Design Recommendations

#### 4.1.1. Phase 1-Focus Groups

1. Comfort is king, except for participants who prioritize accuracy. Participants preferred non-contact solutions like tracking pads and standalone mobile applications over head-mounted devices.
2. Embrace the home sleep setting. Participants were interested in how different environmental factors impacted their sleep and expected apps to incorporate these features to provide actionable feedback rather than just a score.
3. Consider integrating with wellness products and strategies that patient-consumers already use. Participants found many of the demonstrated functionalities duplicative and indicated an interest in integrating CSTs+AI with products they already use, such as fitness trackers, smart devices, and personal health apps.

#### 4.1.2. Phase 2-Usability Study

1. Participants desired feedback about sleep cycles.
2. Other features that participants are interested in are graphs/visuals, interaction from apps, ease of use, aids to help with falling asleep, and alerts of any detected abnormalities.
3. Participants also appreciate the ability to personalize CST+AIs.

### 4.2. Limitations

There are several limitations to this study. Firstly, the choice of sources to identify CSTs may not have captured all available tools. Recruitment was completed using social media, where there could also be sampling bias. Users of social media platforms may not be as concerned with technology privacy and security issues. Although virtual focus groups allowed us to expand the participant pool geographically, the requirement to use a computer and Zoom limited our recruitment. Participation with cameras-on was variable and internet connections and sound quality sometimes varied. The inclusion criteria of a branded smartphone also limited the participant pool. Additionally, the usability study included six participants and only five were able to fully participate. This smaller sample size limits the generalizability of the results. However, the inclusion of five participants completing Part II meets recommended standards for usability research [22]. Due to COVID-19 limitations, this was a home-use study and participants were provided detailed instructions. Without direct observation, there may have been variability in how individuals used the technologies.

Lastly, we used the PSQI, a self-rated and subjective questionnaire about sleep quality, to determine inclusion criteria. Previous studies have shown weak or inconsistent correlations between the PSQI and objective sleep measures, such as PSG.10 The score can range from zero to 21, with a higher score indicating worse sleep quality. For our sample, PSQI scores ranged from 5–18, increasing our confidence that participants did experience difficulties with sleep.

## 5. Conclusions

This study provides insight into user perceptions of CSTs+AI based on focus groups and usability studies. CSTs+AI can be a valuable, affordable, and convenient starting tool for people who have issues or concerns with sleep and want more information. Although there is room for improvement in use, accuracy, and design, CSTs+AI are useful for providing data about the quantity and quality of sleep from the convenience of one’s own bed. There is a need to integrate CSTs with evidence-based sleep self-management tools to provide more tailored advice to the individual. Leveraging AI in a more robust manner could address this need given personal differences in what constitutes optimal sleep. CSTs+AI can empower patient-consumers with information to make better-informed decisions about care with their clinicians. Future work needs to investigate how to integrate CST+AI data into primary care clinical workflows in order to reduce sleep health disparities.

## Figures and Tables

**Table 1:** Consumer sleep technologies using artificial intelligence.

Consumer Sleep Technology Using AI	Device Name (Vendor)
Head-Mounted Devices (HMD)	Beddr (Hancock Medical, Inc., Mountain View, CA, USA) * Dreem Headband (Dreem, Paris, France) Muse S (InteraXon Inc., Toronto, ON, Canada)
Tracking Pads	Sleep Analyzer (Withings, Nanterre, France) * Sleep Number (Sleep Number, Minneapolis, MN, USA) ** Smart Bed (Eight Sleep, New York, NY, USA) **
Standalone Mobile Applications	Do I Snore or Grind (SleepScore Labs, Carlsbad, CA, USA) Sleep Cycle (Sleep Cycle AB, Stockholm, Sweden) * Sleeprate (Sleeprate Tel Aviv, Israel) Sleep Score (SleepScore Labs. Carlsbad, CA, USA)
Responsive Devices	Smart Nora (Smart Nora, Inc., Toronto, ON, Canada)

Consumer sleep technologies using artificial intelligence that were included in this study, organized by technology category.* Used in Phase 2 only. ** Excluded from this study due to storage and shipping limitations.

## Data Availability

On request.

## Abbreviations

The following abbreviations are used in this manuscript:

AI	Artificial Intelligence
CST	Consumer Sleep Technology
CSTs+AI	Consumer Sleep Technologies using Artificial Intelligence
HMD	Head-Mounted Device
PGHD	Patient-Generated Health Data PSG: Polysomnography
PSQI	Pittsburgh Sleep Quality Index SP: Study Participant
SP	Study Participant

## Appendix A. Interview Guides

Focus Group Questions

1. Have you heard about artificial intelligence?
2. How about its applications in healthcare?
3. When you hear about artificial intelligence technology being applied to improving sleep what does that bring to mind?
4. What sleep technologies have you tried, if any?
  - What did you think about these?
  - How did AI play a role in this technology?
5. If you could design a sleep technology, what would you want the device to do?

Following a video demonstration for each technology type:

1. Who do you think would benefit from this technology?
  - What differentiates these from other products?
2. How would they benefit?
  - What features and why?
3. What concerns might there be about this technology?
  - How could these products be improved?
4. Which if any of these technologies do you think you’d be likely to try?
  - Why?

Semi-Structured Interview Questions

1. Have you tried any consumer technologies? If so, what kinds?
2. Before using the technologies, what were your expectations?
3. What would you use sleep technologies for?
4. How easy or difficult was it to use each technology?
5. What did you enjoy about each technology?
6. How do you think these products could be improved?
7. What concerns do you have about these technologies?
8. How do you think people with sleep disorders can benefit from consumer sleep technologies?
9. If you could design a consumer sleep technology, what features would you like it to have?
10. If you would continue using any of these technologies, which one(s) and why?

## Appendix B

**Table A1:** The codebook and definitions generated from a qualitative thematic analysis of the four focus groups in phase one. Major themes in all capitalized letters. Subthemes include an abbreviation of the major theme, a colon, and the subtheme.

Code	Code Definition
AWARENESS_OF_AI	to capture the conversation at the beginning of the focus group, related to participants’ awareness of the use of AI in society in general, in healthcare, and in sleep
ATTRIBUTE	functions or qualities about a product, device, object, item, or strategy
ATTR:Accuracy	information or readings from the product, device, or strategy affected by it falling off, moving around, missing data, lack of trust in the technology, interference by other entities
ATTR:Comfort	how it feels on user, if it interferes with sleep or wakes user up
ATTR:Cost	direct and indirect costs associated with using or purchasing a device, or seeking medical care, medical insurance, or filling prescriptions for pharmaceuticals or devices
ATTR:Ease_of_Use	easy to use interface, able to quickly obtain started, time required to obtain insights from the technology also, related to how the device is charged or powered, gets software updates
ATTR:Adhesive	any mention of the adhesive component of a device
ATTR:Enhancing_Sleep	related to senses of smell, touch, sound, light
ATTR:Feedback	explanations (provides explanations for quality of sleep, patterns in data, or abnormalities in data); summaries (provides summaries of patterns and trends in sleep); recommendations (provides feedbacks, tips, or recommendations for sleep)
ATTR:Guided_Meditation	provides guidance on deep breathing, relaxation, or mental wellbeing
ATTR:Health_Tracking	feature that helps user track aspects of health including sleep (hours slept or wake/sleep times), fitness, dietary, mental wellness, other (e.g., menstrual cycle)
ATTR:Hygiene	how the device can withstand multiple uses or cleaning; cleanliness of the device
ATTR:Integration	whether it can be integrated with the consumer’s other technologies or devices (e.g., FitBit or other health tracking apps)
ATTR:Need_for_Testing	expressed need for evidence, information, research, or proof about the product
ATTR:Personalization	user can decide to customize product, how device is used, or product is customized to the user
ATTR:Privacy	whether the information collected by the product will shared with others
ATTR:Safety	unintentionally hurting self or others with physical device due to movement; radiation from electronic devices, parts, or components (e.g., Bluetooth) that may cause cancer or other side effects or illnesses
ATTR:Utility	usefulness in discussions with doctor, in making treatment decisions, or usefulness in making changes to sleep habits or routines, detection or diagnosis of sleep problems
ATTR:Other	does not fit in the list above
COMPARISON	When the person discusses the pros/cons between two different products or devices.
HEALTH_CONDITIONS	health diagnoses or conditions, such as sleep disorder, anxiety, diabetes, heart conditions, insomnia
LOCATION	the physical location or placement of the object, device, or product, or where the product or mobile application’s will be used
LOC:DeviceBody	and attached to or on body
LOC:DeviceBody:Arm	
LOC:DeviceBody:Chest	
LOC:DeviceBody:Head	
LOC:DeviceBody:Leg	
LOC:DeviceNonbody	device NOT attached to nor on body
LOC:Place:Clinic	Sleep study centers, and research centers
LOC:Place:Home	used in the customer/patient’s primary residence
RELATIONSHIPS	a tag used to identify if a participant comments on relationships with other individuals
REL:Child	
REL:Clinician	physicians, nurses, sleep researchers, etc.
REL:Partner	
REL:Other	for example parents or pets
REL:Self	
SENTIMENT	ideally, every utterance mentioning a device will have a sentiment coded with it
Negative	
NEG:Concern	(-) participant demonstrates worry or anxiety or hesitation about a product or device or feature
NEG:Disapproval	(--) participant demonstrates they do not wish to try or use a product or device or feature
Neutral	participant may find a product or device or feature acceptable but does not explicitly demonstrate concern or interest
Positive	
POS:Interest	(+) participant demonstrates curiosity, wanting to know more about a product or device or feature
POS:Approval	(++) participant demonstrates they would want to try or use a product or device or feature
ST	(sleep therapeutics) a physical item, object, device, or product used in the course of improving sleep
ST:Activity_Band	a wrist band, watch, or other device worn on the body to track components of health (separate from the trackers presented in the focus group)
ST:App	not related to the sleep apps presented during the focus group. this could include music apps, such as Spotify or Pandora or iTunes, podcast apps
ST:CPAP	continuous positive airway pressure therapy and equipment
ST:Eye_Mask	fabric covering worn over the eyes during sleep to prevent light-related disturbance
ST:Headband	objects worn on or around the head, presented during the focus group
ST:Medications	prescribed by a medical professional, over the counter, or recreational
ST:Noise_Machine	white noise machines
ST:Sleep_Apps	the sleep related apps presented during the focus group
ST:Sleep_Pads	the mattress pad presented during the focus group
ST:Snoring_Device	Smart Nora device, presented during the focus group
ST:Other	mouth guards, video recorders, blue light glasses
TEMPORAL	
TEMP:CurrentPast	current or prior use of a sleep therapeutic
TEMP:Future	envisioned or predicted use of a sleep therapeutic
